# H-mode dithering phase studies on ST40

**DOI:** 10.1098/rsta.2021.0225

**Published:** 2023-02-20

**Authors:** Yasmin Andrew, James Bland, Peter Buxton, Alexei Dnestrovskij, Mikhail Gryaznevich, Eun-jin Kim, Michele Romanelli, Marco Sertoli, Paul Thomas, Jari Varje

**Affiliations:** ^1^ Blackett Laboratory, Imperial College London, London SW7 2AZ, UK; ^2^ Tokamak Energy Ltd., 173 Brook Drive, Milton Park, Abingdon, UK; ^3^ Fluid and Complex System Research Centre, Coventry University, Coventry CV1 2TT, UK

**Keywords:** H-mode, pedestal, edge plasma, fusion, plasma physics, L-H transition

## Abstract

The dithering H-mode phase, characterized by oscillations, is generally observed at input power values close to the L-H transition power threshold and low plasma collisionalities (low electron density and/or high plasma temperature). Measurements to characterize the dithering phase are presented for the low aspect ratio, high magnetic field tokamak, ST40. The dithering phase oscillation frequency is observed between 400 and 800 Hz and demonstrates an inverse relationship with core plasma density. Dithering phase H-modes are documented across a nonlinear, low-density power threshold operational space, with signature low- and high-density branches. The minimum power threshold for dithering H-mode access is measured at a core, line average electron density of 4.7(±0.5) × 10^19^ m^−3^, close to a predicted value of 4.1(±0.4) × 10^19^ m^−3^ from multi-machine studies. ASTRA calculated values of power coupled to the ion species, at the dithering H-mode transition, exhibit a similar nonlinear dependence on density. This analysis points to the important contribution of the ion thermal channel to the L-H phase transition. The low-frequency plasma density and D-alpha dithers appear to be accompanied by sudden bursts of magnetohydrodynamic (MHD) activity. A simple model is tested to demonstrate a possible scenario of self-regulation among turbulence, zonal flows, pressure (density) gradient and MHD activities.

This article is part of a discussion meeting issue 'H-mode transition and pedestal studies in fusion plasmas'.

## 1. Introduction

The transition from a low confinement mode (L-mode) to a high confinement phase (H-mode) has been documented in tokamak plasmas since 1982 [1,2] and remains a promising operational regime for next-step magnetically confined fusion devices. The L-H transition is a phase change that occurs when sufficient heating power is applied to the plasma system to reach a threshold input power, *P*_th_. Various tokamaks have documented a dithering phase following the initial transition from L-mode, in which there is a sequence of L-H-L transitions or transitional edge localized modes (ELMs) at values of heating power close to *P*_th_ [3–7]. Under certain conditions, the dithering phase as well as the so-called LCO or I-phase are observed prior to the transition to H-mode. For instance, Xu *et al.* [8] reported the L mode to I-phase and then back to L transition (L-I-L) transition at the marginal input power very close to the L-H transition threshold power while L-I-H transition for a higher input power. In this paper, given the promixty of I phase to the H-mode, we call I-phase the dithering H-mode phase. The dithering H-mode phase provides a unique opportunity to understand the underlying physics and dynamics of both the L-H and H-L phase changes due to the repetitive nature of the dithering cycles, over an expanded window of study time.

Fundamental to the creation of plasma transport barriers and the transition to improved plasma confinement phases is the reduction of electrostatic turbulence by sheared flow. Both larger scale, mean E x B flows (such as, pressure gradient ∇P and external momentum driven) and fine-scale zonal flows (for example, turbulent Reynolds stress driven) are thought to play active, but different roles in turbulence suppression. Zonal flow models, such as Kim & Diamond's [9] predator–prey model, suggest a cyclical interaction between zonal flows, turbulence and mean E x B equilibrium plasma flows. Such predicted cyclical predator–prey interactions have been observed experimentally [10,11] across the L-H transition. The increase in plasma heating in the L-mode phase drives higher levels of plasma turbulence, which in turn excites zonal flows. Before and during the edge transport barrier formation, the mean flow shear is so weak that the zonal flow oscillation may provide the dominant shearing effect, triggering the shear flow turbulence suppression feedback loop of the dithering cycles. As the transport barrier forms, the mean equilibrium flow shear grows with the steepening edge pressure gradient, ∇*P*, which eventually becomes dominant and suppresses the plasma turbulence. This hypothesis is consistent with experimental observations of a modulation of the edge *E_r_* profile and turbulence level in typical limit cycle oscillations, also frequently named as an intermediate phase, I-phase or dithering H-mode phase on a range of different tokamaks [5,11,12]. It has also been noted that low-density plasmas are strongly linked to distinct dithering H-mode phases by Miki *et al*. [13], who speculate that zonal flow damping controls access to the dithering phase in the low-density regime where electron-ion decoupling occurs.

The density dependence of the L-H power threshold has been known for a long time to be non-monotonic on conventional tokamaks, with a minimum power threshold at a given density, *n_e_*_, min_ [14,15]. The power threshold increases on each side of this minimum with increasing and decreasing plasma density, in the so-called low- and high-density branches of the L-H transition. The values of *n_e_*_,min_ have been reported to cover a wide range in different-sized conventional tokamaks in [16], between 2.0 × 10^19^ m^−3^ and 1.5 × 10^20^ m^−3^. The larger tokamaks such as JET and JT60-U populate the lower *n_e_*_, min_ range, medium size tokamaks DIII-D and ASDEX-U lie somewhat higher and the highest values have been measured for the high-field, compact machine, Alcator C-Mod [16]. The authors are not aware of any experimental reports of *n_e_*_,min_ for spherical tokamaks in the literature.

It is also important to note that the fundamental dynamics of the H-L transition may also be quite different from that of the L-H, as the plasma conditions that precede the phase change, such as plasma profiles and turbulence are significantly different in the H-mode phase. There have been relatively few H-L transition and power hysteresis studies [13,14,17], especially in low-density regions, possibly due to the more complicated experimental control requirements to systematically access the back transition. These issues are indirectly addressed in this paper by the data analysis of the experiments on ST40 spherical tokamaks that were performed under unique conditions to enable access to the dithering phase near the transition to the H-mode.

In this paper, the experimental conditions and analysis methods used to study the access to the dithering phase H-mode in the low-density region on ST40 are described in §2. Section 3 describes the experimental characteristics observed for the dithering H-mode phase of the plasmas. The power threshold and temperature analysis are included in §4, along with the power balance calculations for the different plasma species and an extended zonal flow model. The last section comprises a summary of the main results from the study along with planned futurework.

## 2. Experiment

**Figure 1.**
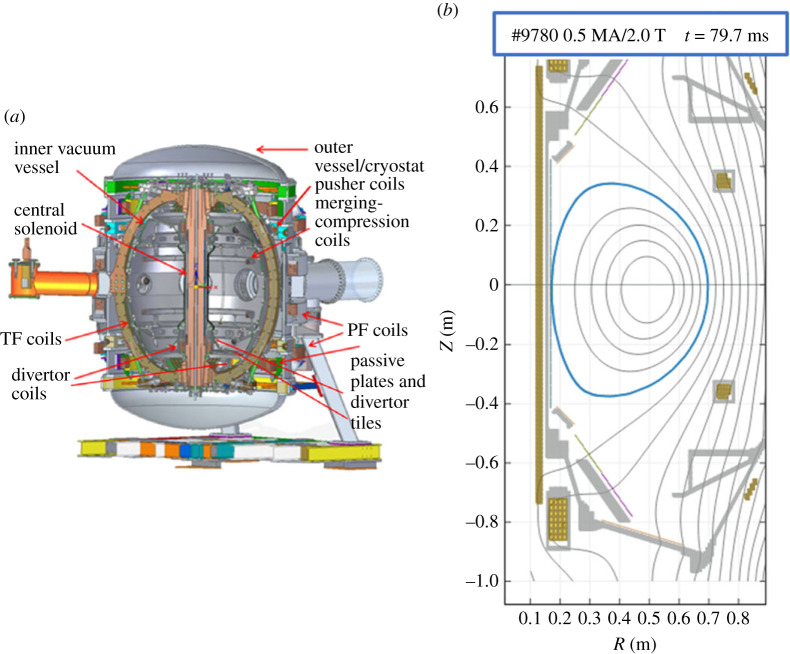
(*a*) Cross-section of ST40 machine. (*b*) Magnetic configuration of example shot #9780 at time, *t* = 79.7 ms, just prior to L-H transition. (Online version in colour.)

**Figure 2.**
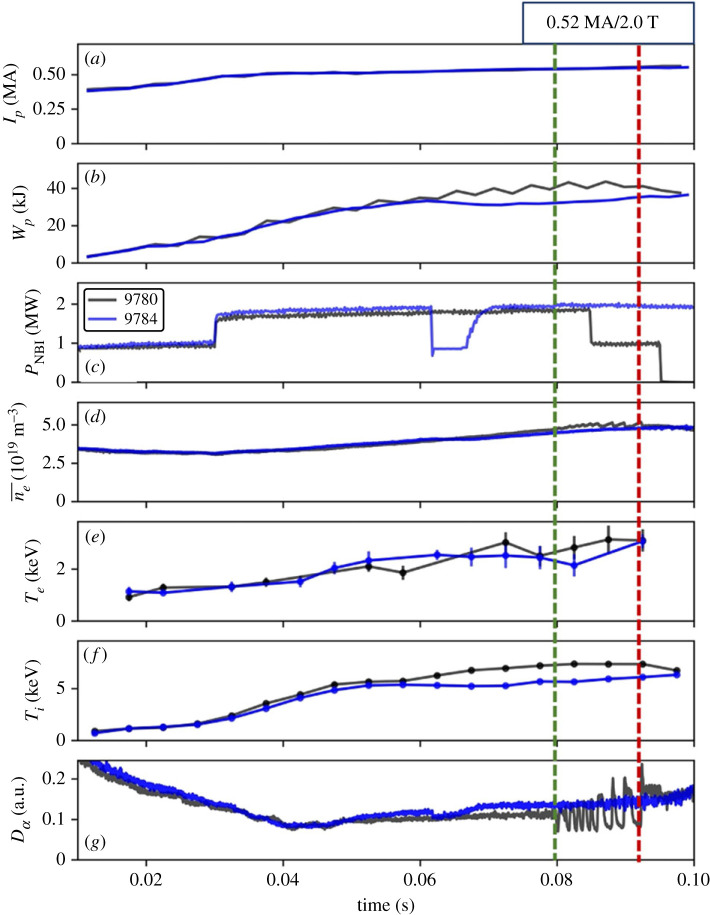
General parameters for shot numbers #9780 and #9784 (*a*) plasma current, *I_p_*, (*b*) plasma energy, *W_p_*, (*c*) NBI power P_NBI_, (*d*) core line average electron density, $\bar{n_{e}}$, (*e*) core volume electron temperature $\bar{T_{e}}$, (*f*) core volume average ion termperature $\bar{T_{i}}$ and (*g*) *D_α_* emission. The vertical dashed lines mark the start (green) and end (red) of the dithering phase. (Online version in colour.)

The dithering H-mode phase results presented in this paper are from the compact, high-field, spherical tokamak, ST40 [18] shown in figure 1*a*, with plasma major radius, *R* = 0.46–0.48 m, and minor radius, a = 0.26 m. A representative magnetic configuration at the start of the H-mode, is shown in figure 1*b* for shot number 9780, at *t* = 79.7 ms prior to the L-H transition. The general plasma parameters for two shots, #9780, which transitioned to a dithering H-mode, and #9784, which remained in L-mode, are compared in figure 2.

Neutral beam injection (NBI) was the only auxiliary heating method used; one of the injectors provided a series of 0.1–0.2 MW incremental power steps over 11–12 ms. All shots included in this study were fuelled with the deuterium NBI in the quasi-stationary plasma current phase and example NBI power time traces are shown in figure 2*c* for the shots #9780 and #9784.

The plasma current typically increased from 0.52 MA to a maximum of 0.56 MA over the window of NBI injection, as shown for the example plasmas figure 2*a*. A plasma density scan was performed for the series of shots in this study at *I_p_*/*B_t_* = 0.52 MA/2.0 T over a range of, $\bar{n_{e}}=3.8-5.2\times 10^{19}{\text{m}}^{-3}$ with plasma elongation between, *κ* = 1.34–1.44, at the L-H transition.

In the core plasma, electron and ion temperatures, $\bar{T_{e}}$ and $\bar{T_{i}}$, are measured using a crystal X-ray spectrometer set to the He-like argon emission spectal region around 4 nm [19] with a time resolution of 5 ms. Typical values of $\bar{T_{e}}$ and $\bar{T_{i}}$ are shown across the transitions for the example shot in figure 2*e* and *f*. This instrument views along a radial line-of-sight through the mid-plane. The $\bar{T_{e}}$ and $\bar{T_{i}}$ measurements shown in figure 2*e* and *f* are averaged values over the emission region of He- and Li-like ions and therefore a lowest estimate of the central plasma temperatures. The last L-mode and H-mode $\bar{T_{i}}$ and $\bar{T_{e}}$ points, taken preceding the forward and back, L-H and H-L transitions have been used in the analysis.

The line averaged core plasma electron density, $\bar{n_{e}}$, is measured with an interferometer along a single radial mid-plane chord with a time resolution of 40 µs shown in figure 2*d*. The plasma-stored energy, *W_p_*, is evaluated from the reconstructed EFIT [20] magnetohydrodynamic (MHD) plasma equilibrium. Both in-vessel and external sensors are used to measure the ST40 magnetic fields [21].

## 3. Dithering phase characterization

The L-H transition for these plasmas is characterized by a transition from L-mode to a dithering H-mode phase. The *D_α_* emission and $\bar{n_{e}}$ time traces clearly show a quiescent L-mode phase, followed by a sharp transition into a dithering H-mode phase, with characteristic drop in *D_α_* intensity and rise in $\bar{n_{e}}$. Following previous work in the field, the L-H transition is taken as the transition, after which the plasma enters the dithering phase [15,22,23]. The H-L transition is considered in this study to be the final point, following which the plasma exits the (dithering) H-mode and transitions back to and remains in L-mode.

The temporal evolution of two shots with comparable *I_p_*, *B_t_* and core plasma L-mode $\bar{n_{e}}$ and $\bar{T_{e}}$ have been compared to characterize an example dithering H-mode phase. One of the shots (#9784) features a notch in the auxiliary heating power since one of the NBI injectors dropped to 0 MW at *t* = 61.6 ms for 8 ms. The short reduction in power was sufficient to prevent the plasma from transitioning to the H-mode and provides a very useful L-mode reference shot under very similar vessel and plasma conditions. The L-H transition for shot #9780 occurs at *t* = 79.8 ms, at which time a difference in plasma energy of Δ*W_p_* = 7.3 kJ, $\bar{\Delta n_{e}}=0.1\times 10^{19}\;{\text{m}}^{-3}$ and $\bar{\Delta T_{i}}=1.8\;\text{keV}$. The core $\bar{T_{i}}$ is observed to increase in the L-mode segment of #9780 relative to the comparison plasma, approximately 20 ms before the transition to the dithering phase. The start of the divergence in core $\bar{T_{i}}$ appears to coincide with the timing of the notch in the additional heating in the L-mode shot #9780. The plasma remains in the dithering H-mode phase for the remainder of the final auxiliary power step of 12 ms; over this time a maximum difference of *W_p_* = 9.9 kJ and $\bar{n_{e}}=0.4\times 10^{19}\;{\text{m}}^{-3}$ develops, as shown in figure 2*b*,*d*, displaying the enhanced energy and particle confinement characteristic of H-mode development. From figure 2*f*, the corresponding maximum difference in $\bar{T_{i}}$ over the dithering H-mode phase remains at $\bar{\Delta T_{i}}=1.8\;\text{keV}$.

**Figure 3.**
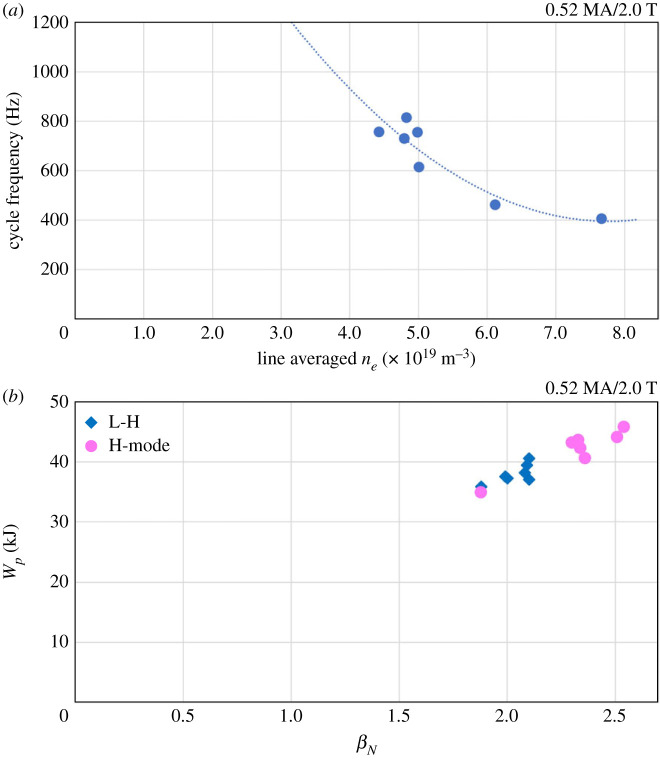
(*a*) Average dithering cycle oscillation frequency as a function of core plasma, line average electron density, $\bar{n_{e}}$, at the final H-L transition of each shot. The dotted line represents a fit to the data. (*b*) Maximum values of plasma energy, *W_p_*, and normalized beta, *β_N_*, at the L-H transition and in the dithering H-mode phases. (Online version in colour.)

The frequency of the H-mode cycles of the dithering phase are found to decrease with core density, as shown in figure 3*a*, in which the average number of cycles over the dithering time window has been considered. The cycle frequency is observed to vary between 400 and 800 Hz over the $\bar{n_{e}}$ range measured in the last dithering cycle of each plasma. The maximum values of plasma energy and normalized beta have been measured to lie between *W_p_* = 35–39.5 MJ and *β*_N_ = 1.9–2.1 at the L-H transition, compared with peak values in dithering H-mode of *W_p_* = 35–45.6 MJ and *β*_N_ = 1.9–2.54. The data presented in figure 3*b* clearly show the L- and H-mode segments of the shots to be differentiated by the associated values of stored *W_p_* and *β_N_*.

## 4. Roles of the ion heating channel and self-regulating parameters

Following conventional analysis of experimental values in the field, the loss power, *P_L_*, is defined at:

4.1 $$P_{L}=P_{IN}-\frac{\text{d}W_{p}}{\text{d}t},$$

where *P*_IN_ is the sum of the input power, which in these plasmas are ohmic and NBI, and d*W_p_*/d*t* is the rate of change of stored plasma energy. The value of $\text{d}W_{p}/\text{d}t$ is typically less than 10% of the total input power for these shots. The L-H and H-L transition threshold powers, *P*_th_, have been taken as the values of *P_L_*, at the time of the forward and back transitions, defined as the first and final transition into and from the dithering phase, as described above.

**Figure 4.**
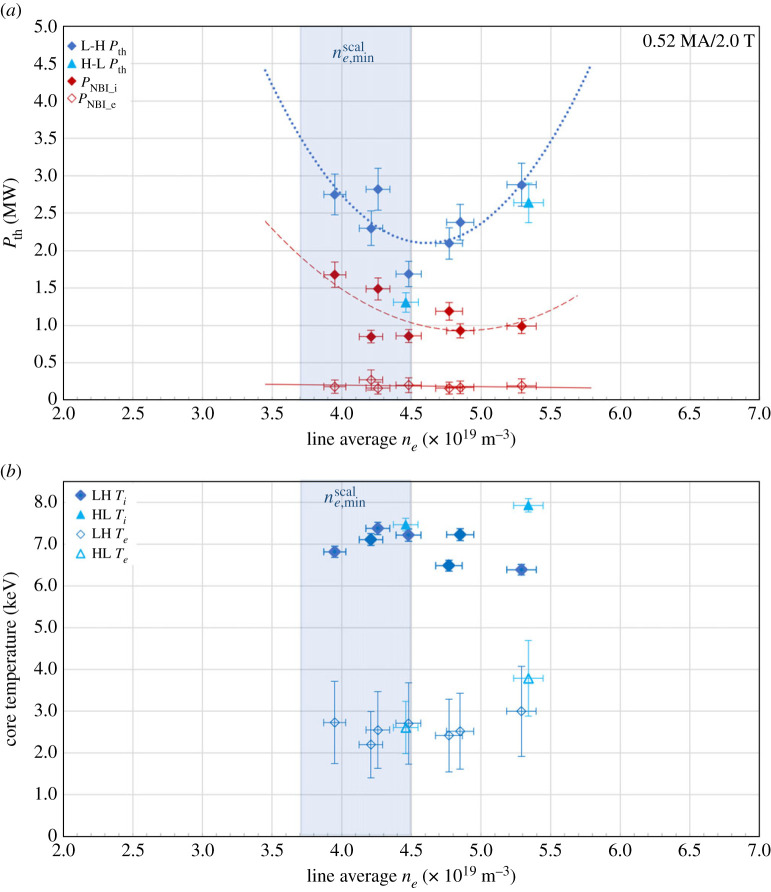
(*a*) Power threshold, *P*_th_, for L-H and H-L transitions and ASTRA calculated values of NBI power coupled to ions and electrons, *P*_NBI_i_ and *P*_NBI_e_, at the L-H transition as a function of core plasma, line average $\bar{n_{e}}$. The lines represent polynomial fits to the datasets. The vertical shaded region represents the predicted region of $n_{e,\text{min}}^{\text{scal}}=4.0\times 10^{19}$ (±10%) m^−3^. (*b*) Core plasma, volume average $\bar{T_{i}}$ and $\bar{T_{e}}$, as a function of core $\bar{n_{e}}$, for the L-H and H-L transitions. (Online version in colour.)

The values of the L-H and H-L *P*_th_ for seven plasmas (#9780, #9781, #9783, #9787, #9831, #9835 and #9892) are plotted in figure 4*a* as a function of $\bar{n_{e}}$, along with the polynomial fit to the L-H *P*_th_ data. These results demonstrate a nonlinear dependence of *P*_th_ on $\bar{n_{e}}$, characteristic of the well-known low-density region of H-mode access. The minimum value of *P*_th_ from the fit to the data lies in the region of core $\bar{n_{e,\text{min}}}=4.7(\pm 0.5)\times 10^{19}\;{\text{m}}^{-3}$.

The vertical shaded area in figure 4*a* indicates the predicted value of $n_{e,\text{min}}^{\text{scal}}$ with an assumed error of (±10%), from the expression derived by Ryter *et al.* [14] for a multi-machine deuterium plasma dataset,

4.2 $$n_{e,\text{min}}^{\text{scal}}\approx 0.7I_{p}^{0.34}B_{t}^{0.62}a^{-0.95}{\left(\frac{R}{a}\right)}^{0.4},$$

in 10^19^ m^−3^, where *I_p_* in in MA, *B_t_* is in *T*, *a* and *R*, are in m. Ryter *et al*. [14] suggest that the minimum threshold density is determined by the collisional coupling between electron and ion thermal energy channels, such that below a given plasma density, it is not large enough to fully equilibrate *T_e_*, *T_i_* thermal fluxes, even in the relatively cooler edge plasma region.

The experimental value of $\bar{n_{e,\text{min}}}$ measured in this study is found to agree remarkably well with $n_{e,\text{min}}^{\text{scal}}=4.1(\pm 0.4)\times 10^{19}{\text{m}}^{-3}$. This is attributed to the relatively high values of *I_p_* and *B_t_* accessible in ST40, along with the hot-ion mode plasma intrinsic separation of the ion and electron heating channels.

The heating power coupled to the ion, *P_NBI_i_*, and electron, *P_NBI_e_*, plasma species have been analysed using a time-dependent power balance calculation with the ASTRA code [24,25] in MW. The calculated values of *P_NBI_i_* and *P_NBI_e_* are compared with the values of *P*_th_ in figure 4*a*. The behaviour of *P_NBI_i_* exhibits a similar trend to *P*_th_, varying nonlinearly with $\bar{n_{e}}$, while the values of *P_NBI_e_* decrease linearly with increasing $\bar{n_{e}}$. These results provide further evidence that the ion heating channel dominates the observed *P*_th_ dependence on $\bar{n_{e}}$ in this low-density region.

The corresponding values of core $\bar{T_{i}}$ and $\bar{T_{e}}$ at the L-H and H-L transitions are shown as a function of core $\bar{n_{e}}$ in figure 4*b*. The values of line averaged ion temperature measured using the He-like argon emission, $\bar{T_{i}}$ exhibit a nonlinear dependence on $\bar{n_{e}}$ with the maximum $\bar{T_{i}}=7.4(\pm 0.1)\text{keV}$ at $\bar{n_{e}}=4.3\times 10^{19}{\text{m}}^{-3}$. The measured values of core $\bar{T_{e}}$ decrease from a maximum of 3.0 (±0.73) keV to minimum of 2.2(±0.75) keV across the $\bar{n_{e}}$ scan. The ratio of ion to electron temperature at the L-H transition is in the range, $\bar{T_{i}}/\bar{T_{e}}=2.1--3.2$ = , since the ion heat channel is dominated by the NBI contribution and weak collisional energy transfer from the ion to electron energy channel. The difference in $\bar{T_{i}}/\bar{T_{e}}$ at the L-H transition is reduced at the higher values of $\bar{n_{e}}$, which could be attributed to increased plasma collisionality in the high-density branch of the *P*_th_ curve.

The H-L transition for two of the plasmas (#9781 and #9831) occurred within the NBI heating phase and were used to determine the H-L *P*_th_, also plotted in figure 4*a*. These H-L transitions appear to be triggered by the elevated values of dithering phase H-mode plasma density at the limited input power. The H-L *P*_th_ is measured to be up to 10% lower than the corresponding L-H *P*_th_. For the observed H-L transitions, the values of $\bar{T_{i}}$ and $\bar{T_{e}}$ included in figure 4*b* are observed to be comparable with the forward transition values, for the lower plasma density, $\bar{n_{e}}=4.3\times 10^{19}{\text{m}}^{-3}$. At the higher plasma density, while $\bar{T_{e}}$ values at the forward and back transitions agree within the uncertainities, the H-L transition *T_i_* is measured to be 1.2 times higher than for the L-H transition. The transition from H-mode to L-mode is less well controlled and therefore more difficult to access than the forward transition. While this limited dataset of two points indicates a small amount of hysteresis in power and differences in ion temperature observations over the density scan, more data are required over the $\bar{n_{e}}$ region to evaluate any differences fully.

**Figure 5.**
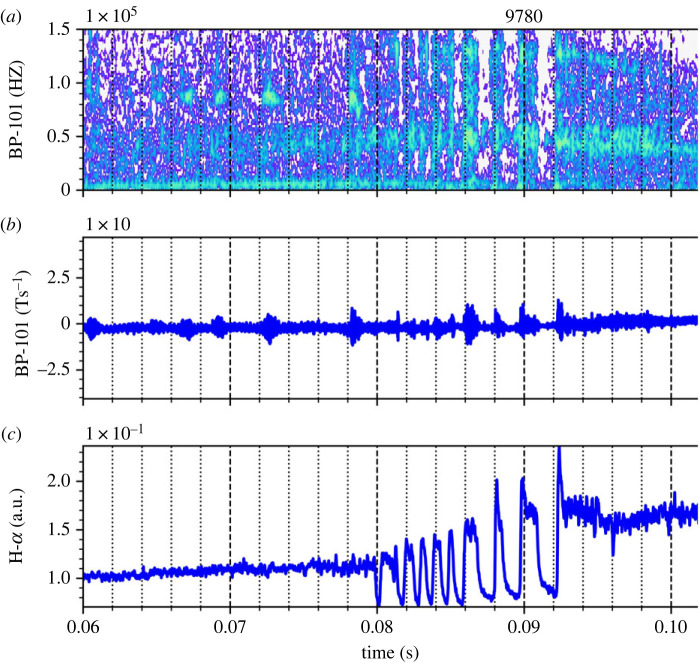
(*a*) Magnetospectrogram of the poloidal magnetic field for #9780 and the corresponding (*b*) d*B_p_*/d*t* and (*c*) *D_α_* emission. (Online version in colour.)

The physical origin of the observed dithering is not entirely clear given no experimental measurements of edge plasma turbulence, rotation, radial electric field, etc. Although the dithering cycles may be due to self-regulation between self-generated zonal flows and turbulence [8], it seems that there are additional players involved in self-regulation since there is some indication of the involvement of MHD activity in the cycles. As an example, figure 5 shows a magnetospectrogram (*a*), d*B_p_*/d*t* (*b*) and D-alpha (*c*) for shot #9780. The dominant frequency of the MHD activity can be seen *f* < 1 kHz. The observed abrupt occurrences of large fluctuations in d*B_p_*/d*t* are associated with a sudden rise in D-alpha and a sudden collapse of $\bar{n_{e}}$. These allude to the possibility of the involvement of low-frequency MHD activities due to a steep density or pressure profile.

**Figure 6.**
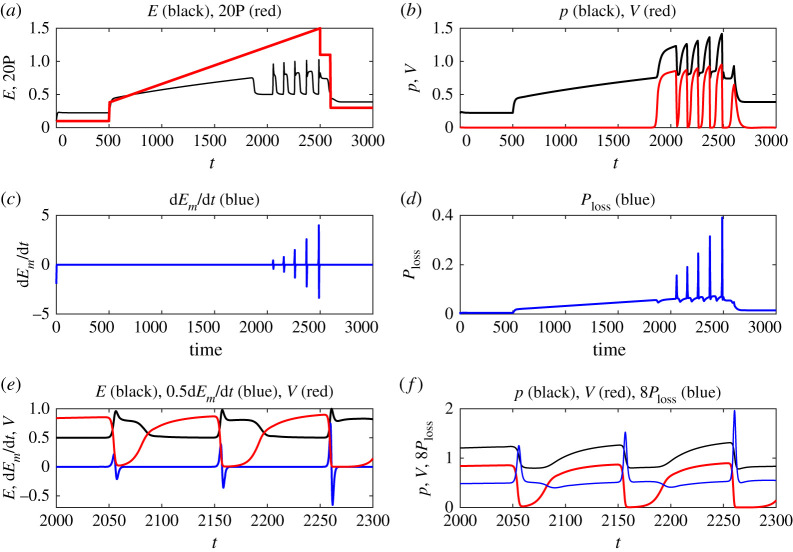
Results from the toy model in (a1)–(a4) for the parameter values *d* = 0.1, *d_m_* = 0.05, *λ* = 5, *α* = 0.5, *μ* = 0.25. Time unit is of order 1 µs. The time evolutions of (*a*) turbulence *E* and input power *P* = 0.005 + 0.000028*t*Θ(*t* − 500)Θ(2500 − *t*) + 0.05*t*Θ(*t* − 2500)Θ(2600 − *t*) + 0.01Θ(*t* − 2600), where Θ(x) is the Heaviside function, (*b*) pressure gradient *p* and E x B zonal flows *V*, (*c*) d*E_m_* /d*t*, (d) powerloss *P*_loss_ = *P* − (d*p*/d*t*). Zoomed-in views in (*e*) and (*f*). (Online version in colour.)

Assuming electrostatic turbulence in the L-mode, including MHD pressure gradient-driven instabilities in [8] would lead to an extended model (a1)–(a4) provided in appendix A, quite similar to the ELM model of Lededev *et al*. [26]. As noted in appendix A, in this model, zonal flows *V* are generated by turbulence through the first term *αEV* on the right-hand side of equation (a3) while damped directly by *E_m_* as well as collisional damping. The example time evolutions of the mean ion pressure gradient, *p* (proportional to mean density gradient by assuming constant temperature), MHD activity, *E_m_*, and turbulence amplitude, *E*, together with input power, *P*, are shown in figure 6, μs (see appendix A and [26] for non-dimensionlization). To mimic the experiment, *P* is taken to increase up to *P* = 0.075 at = 2500 and then ramped twice down to *P* = 0.015 at *t* = 2600, as shown in figure 6*a*. Model parameters (see appendix A) are chosen to ensure the appearance of dithering shortly after the L-H transition.

Figure 6*a*,*b* shows that as turbulence amplitude *E* grows, zonal flows *V* are amplified starting around *t* ≈ 1800, marking the L-H transition at *P_cr_* ≈ 0.7. However, when *V* grows to a sufficiently large amplitude, it damps *E*, causing a certain phase shift between the oscillations of *E* and *V*. That is, *V* and *E* do not share a similar phase. At the same time, turbulence regulation by *V* leads to steeping of the edge pressure gradient *p*, causing MHD activity *E_m_* in figure 6*f*, which damps not only *p* but also *V* (through the damping term *E_m_ V* in equation (a3)). MHD activity is also manifested as a sudden spike in $\text{d}E_{m}/\text{d}t$ in figure 6*c* and powerloss $P_{\text{loss}}=P-(\text{d}p/\text{d}t)$ in figure 6*d*. The maximum power at *P *= 0.075 at *t* = 2500 is about 7% above *P_cr_* ≈ 0.7. Self-regulation among *E*, *V*, *E_m_* and *p* (mean shear flow ∝ *p*^2^) can be seen more clearly in zoomed-in views in figure 6*e*,*f*. Note that because of the damping of *V* by magnetic activity, the self-regulation between turbulence *E* and zonal flows *V* is less evident than what was observed in [9]. If *E_m_* = 0 were used for the parameter values used in figure 6, dithering would not appear. In summary, this toy model provides a simple explanation of the observed cycles of the dithering H-mode phase. Further comparative and quantitative studies are planned as turbulence measurements on ST40 become available.

## 5. Conclusion

A plasma density scan has been performed on the ST40 that shows a minimum *P*_th_ for access to a dithering H-mode phase. All the plasmas remain in the dithering H-mode phase before transition back to L-mode due to the available power margin remaining close to the L-H *P*_th_ and in the absence of plasma density feedback control.

The core electron density at which the minimum *P*_th_ is measured has been observed at $\bar{n_{e,\text{min}}}=4.7\times 10^{19}{\text{m}}^{-3}$. This value is very close to the predicted values of $n_{e,\text{min}}^{\text{scal}}=4.1\times 10^{19}{\text{m}}^{-3}$, demonstrating the importance of *I_p_* and *B_t_* in governing the *P*_th_ on n_e_ in the nonlinear, low-density region. The calculated values of *P_NBI_i_* show a similar nonlinear dependence on core $\bar{n_{e}}$ at the L-H transition, while *P_NBI_e_* is observed to have a weak negative linear dependence over the density range scanned. These results indicate the ion thermal channel plays a key role in accessing the H-mode.

The line averaged core ion temperature just prior to the L-H transition has a peak value of $\bar{T_{i}}=7.4\;(\pm 0.1)\text{keV}$ and decreases with increasing $\bar{n_{e}}$ while the line averaged core plasma $\bar{T_{e}}$ values are observed to be fairly constant across the scan. The core $\bar{T_{i}}$ remains a factor of 2–3 times higher than $\bar{T_{e}}$ for all shots, due to the dominance of the ion heating through NBI and very little collisional heating between the two species.

Data for the power threshold for H-L transition have been included for two plasmas in this study and indicate a maximum of 10% hysteresis in the power threshold. Experiments are planned in forthcoming campaigns to carefully study and evaluate hysteresis in the ST40 H-mode.

An extended self-regulatory limit cycle model has been developed to include turbulence amplitude, zonal flows, pressure gradient and MHD activity. This model reproduces the qualitative features of the dithering H-mode cycles well and points to the possible contribution of low-frequency MHD, due to a steep density or pressure profile, to the L-H-L cycle in the dithering phase of the plasmas.

It will be interesting to include the effect of additional terms in forthcoming experimental H-mode and L-H/H-L transition investigations and power balance calculations; factors such as NBI shine through losses, fast ion losses due to charge exchange and ion orbit processes and radiated power. In addition, experiments to study the role of the edge, pedestal and SOL plasma parameters are foreseen on ST40 with an upgraded set of Charge Exchange Recombination Spectroscopy and Thomson Scattering diagnostics. Dedicated experiments are also planned with the updgraded diagnostic suite to build and expand the H-mode and L-H/H-L transition database with different plasma configurations.

## Data Availability

The ASTRA code used is explained in detail in [24] and [27] for a more recent configuration. The data used in this article are from the ST40 experimental campaign run during January 2022 at Tokamak Energy Ltd, UK. The data in the graphs within this paper are not held in a public repository, however, the corresponding author will be happy to discuss ways to access the data upon reasonable request.

## Appendix A

The toy model used for figure 6 consists of the evolution of the dimensionless pressure gradient *p*, turbulence amplitude *E*, E x B shear flows (zonal flows) *V*, magnetic fluctuation amplitude *E_m_*, as follows:

A 1 $$\frac{\text{d}p}{\text{d}t}=P-(dE+d_{m}E_{m})p,$$

A 2 $$\frac{\text{d}E_{m}}{\text{d}t}=\lambda (p-1-aV^{2})E_{m},$$

A 3 $$\frac{\text{d}V}{\text{d}t}=\alpha EV-(\mu +E_{m})V,$$

A 4 $$\;\text{and}\;\frac{\text{d}E}{\text{d}t}=E(p-E-V^{2}).$$

Here, *P* is roughly proportional to input power [24]; *d*, *d_m_* , *λ*, *α*, *μ* are positive constant; *a* > 0 when poloidal flows *V* mainly damp *E_m_* via shearing while *a* < 0 when they help MHD instability via centrifugal forces. In this model, zonal flows *V* are driven by turbulence *E* while being damped by magnetic field *E_m_* in addition to collisional damping *μ*. Although in [26], E x B shear flows *V* were not referred to as zonal flows, they are assumed to be generated from turbulence *E* in the model by the first term *αEV* on the right-hand side of equation (A 3), similarly to zonal flows driven by turbulence in [26]. However, there is no experimental measurement of the ion velocity from the experiments, and the identification of V is thus open to discussion.

We note that equations (A 1)–(A 4) are non-dimensionalized such that time *t* is in the units of ${\left[\left(c_{s}/\rho_{s})k\rho_{s}({\Delta^{4}}_{c}/{\rho^{2}}_{s}{L^{2}}_{p}\right)\right]}^{-1}$ where *c_s_* = (*T_e_*/*m_i_*)^1/2^ is the ion sound speed, *ρ_s_* = *c_s_*/*ω_ci_*, *ω_ci_* is the ion cyclotron frequency and *k* and *Δ_c_* are the poloidal wave number and radial correlation length of the turbulence, respectively (see [24] for details). Obviously, parameter values depend on the characteristics of plasmas and turbulence. For ST40 edge plasmas, there are presently no measurements of turbulence characteristics to be able to estimate the result of the parameters and the time unit.

The parameter values for figure 6 are *d* = 0.1, *d_m_* = 0.05, *λ* = 5, *α* = 0.5, *μ* = 0.25. To mimic the power ramping in figure 2*c*, we use *P* = 0.005 + 0.000028*t*Θ(*t* − 500)Θ(2500 − *t*) + 0.05*t*Θ(*t* − 2500)Θ(2600 − *t*) + 0.01Θ(*t* − 2600), which increases up to *p* = 0.075 and is then ramped down to *p* = 0.015. Here, Θ(x) is the Heaviside function. In this model, MHD refers to rather low-frequency activities averaged over high-frequency modes (e.g., due to the interaction with high-energy particles). Near the threshold power, E x B shear flows (zonal flows) are approximated by poloidal flows *V*.
